# Radiation‐induced effects on TGF‐β and PDGF receptor signaling in cancer‐associated fibroblasts

**DOI:** 10.1002/cnr2.2018

**Published:** 2024-03-15

**Authors:** Nannan Yang, Turid Hellevik, Rodrigo Berzaghi, Inigo Martinez‐Zubiaurre

**Affiliations:** ^1^ Department of Community Medicine, Faculty of Health Sciences UiT The Arctic University of Norway Tromsø Norway; ^2^ Department of Radiation Oncology University Hospital of North Norway Tromsø Norway; ^3^ Department of Clinical Medicine, Faculty of Health Sciences UiT The Arctic University of Norway Tromsø Norway

**Keywords:** cancer‐associated fibroblasts, ionizing radiation, PDGFR, radiotherapy, signaling, TGF‐β, TGFβR, tumor microenvironment, tumor stroma

## Abstract

**Background:**

Cancer‐associated fibroblasts (CAFs) consist of heterogeneous connective tissue cells and are often constituting the most abundant cell type in the tumor stroma. Radiation effects on tumor stromal components like CAFs in the context of radiation treatment is not well‐described.

**Aim:**

This study explores potential changes induced by ionizing radiation (IR) on platelet‐derived growth factor (PDGF)/PDGFRs and transforming growth factor‐beta (TGF‐β)/TGFβRs signaling systems in CAFs.

**Methods and Results:**

Experiments were carried out by employing primary cultures of human CAFs isolated from freshly resected non‐small cell lung carcinoma tumor tissues. CAF cultures from nine donors were treated with one high (1 × 18 Gy) or three fractionated (3 × 6 Gy) radiation doses. Alterations in expression levels of TGFβRII and PDGFRα/β induced by IR were analyzed by western blots and flow cytometry. In the presence or absence of cognate ligands, receptor activation was studied in nonirradiated and irradiated CAFs. Radiation exposure did not exert changes in expression of PDGF or TGF‐β receptors in CAFs. Additionally, IR alone was unable to trigger activation of either receptor. The radiation regimens tested did not affect PDGFRβ signaling in the presence of PDGF‐BB. In contrast, signaling via pSmad2/3 and pSmad1/5/8 appeared to be down‐regulated in irradiated CAFs after stimulation with TGF‐β, as compared with controls.

**Conclusion:**

Our data demonstrate that IR by itself is insufficient to induce measurable changes in PDGF or TGF‐β receptor expression levels or to induce receptor activation in CAFs. However, in the presence of their respective ligands, exposure to radiation at certain doses appear to interfere with TGF‐β receptor signaling.

## 1 INTRODUCTION

Cancer‐associated fibroblasts (CAFs) are one of the most abundant and essential components among all the stromal cells that reside in the tumor microenvironment (TME). CAFs provide not only physical support for tumor cells, but also play a central role in promoting or hindering tumorigenesis in a context‐dependent manner.^1^ The presence of CAFs in the TME is frequently correlated with expanded angiogenesis, invasion, and metastasis, and thus associated with poorer prognosis in a wide variety of solid malignancies.^2^, ^3^ Besides, CAFs are recognized mediators of immunosuppression in the TME.^4^, ^5^ Of note, recent reports highlight the participation of CAFs in therapy resistance.^3^, ^6^ In the context of radiotherapy (RT), the ultimate role played by CAFs in therapy outcomes remain unresolved.^7^ Although some studies claim that RT have detrimental effects on CAFs by inducing growth arrest and impaired motility,^8^, ^9^, ^10^ others argue that exposing fibroblasts to ionizing radiation (IR) promotes their conversion into a more activated and aggressive phenotype.^11^ Of importance, CAF‐mediated immunosuppressive functions seem to be preserved in post‐radiotherapy settings.^7^, ^12^, ^13^, ^14^, ^15^ Nevertheless, the field is still in the need of further knowledge that can help to better understand CAF responses to radiation, and to elucidate the potential role that CAFs may play in tumor radioresistance.

The transforming growth factor‐beta (TGF‐β) and platelet‐derived growth factor (PDGF) signaling systems are among the most important regulatory pathways of mesenchymal cells differentiation and functions.^16^, ^17^ The PDGF system is assembled from five homo or heterodimeric ligands (PDGF‐AA, AB, BB, CC, and DD) and two receptor tyrosine kinases, PDGFRα, and PDGFRβ, which can dimerize either homologously or heterologously.^18^ Ligand‐binding to PDGFRs induces receptor phosphorylation followed by activation. PDGFRs phosphorylation leads to recruitment of a huge amount of signaling molecules to certain intracellular phospho‐tyrosine residues, that together launched a complete and proper cellular response. The involvement of stromal PDGFRs on tumor growth regulation have been demonstrated in different cancer models.^19^, ^20^ PDGFRβ signaling also play a crucial role in regulating pericytes behavior and function.^21^

Transforming growth factor‐beta (TGF‐β) is a fundamental regulator of tissue repair, embryonic development, and adult tissue homeostasis.^22^ In cancer, TGF‐β functions as an effective inhibitor of early‐stage tumorigenesis but may participate in tumor progression and metastasis at late stages.^23^, ^24^ Moreover, TGF‐β is well‐known to suppress antitumor immune responses^25^, ^26^ and thus constitutes a barrier for successful cancer immunotherapy.^27^, ^28^, ^29^ In the context of radiotherapy, radiation‐induced disturbances on the TME trigger activation and release of matrix‐bound latent TGF‐β, which ultimately contribute to the establishment of an immunosuppressive TME and the activation of epithelial‐mesenchymal transition (EMT) processes in tumor cells.^30^ Hence, several preclinical and clinical studies have indicated the potential for enhanced treatment responses by blocking TGF‐β in combined (RT) strategies.^28^, ^29^, ^31^, ^32^, ^33^, ^34^

TGF‐β, which exist in three different isoforms, is normally available in its inactive form, coupled to either latent TGF‐β binding protein (LTBP) or glycoprotein‐A repetition proteins (GARPs).^35^, ^36^ Upon binding with TGF‐β active form, TGFβRII heterodimerizes with and phosphorylates TGFβRI/ALK5. In the canonical pathway, the activated receptor‐ligand complex results in phosphorylation of the Smad2/3 transcription factors, followed by receptor dissociation of phosphorylated SMADs and complex formation with Smad4, before nuclear translocation and transcriptional regulation of a large number of genes.^37^ As an alternative, TGF‐β can bind to TGFβRII/ALK1/endoglin complexes or TGFβRII/ALK5 complexes to activate Smad 1/5/8 signaling (noncanonical pathway).^38^ Hence, SMAD phosphorylation is a direct read‐out of TGFR activity. Both the PDGF/PDGFRs and TGF‐β/TGFβRs signaling systems play major roles in the regulation of multiple CAFs functions, including cell cycle, cell motility, cell death, extracellular matrix (ECM) remodeling, immunoregulation, metabolic reprogramming, and myofibroblast differentiation.^17^, ^18^, ^20^, ^23^, ^39^

CAFs are recognized as key contributors to tumor initiation and development, and accumulated evidence suggest that they participate in the establishment of therapy resistance. In the context of radiotherapy, several studies performed on clinical specimens have demonstrated clear associations between high expression levels of CAF specific markers, or CAF signature genes, with poor therapeutic outcomes.^40^, ^41^, ^42^ Moreover, preclinical studies suggest that CAFs from different cancer types exert radioprotective effects on tumor cells,^43^, ^44^, ^45^ and some studies claim that radiation exposure is amplifying the intrinsic radioprotective and pro‐malignant effects exerted by CAFs.^46^, ^47^, ^48^, ^49^ However, the mechanisms behind the observed enhanced pro‐malignant features in irradiated CAFs remain incompletely understood.

Fibroblast activation is triggered primarily by the secretion of potent factors derived from epithelial/tumor cells such as TGF‐β, PDGF, and basic fibroblast growth factor (bFGF), resulting in increased proliferation, increased contractility, and expression of activation markers such as αSMA, PDGFRα/β, fibroblast activation protein (FAP), podoplanin (PDPN), and desmin.^1^, ^50^, ^51^ Given the pivotal role of TGF‐β and PDGF receptor signaling in fibroblast/CAF activation and in the acquisition of their pro‐tumorigenic properties, in this study we have explored if radiation exposure translates into relevant changes in TGF‐β /PDGF receptor expression and/or signaling in CAFs.

## 2 MATERIALS AND METHODS

### 2.1 Human material, tumor fibroblast isolation, and cell culturing

Human CAFs were isolated from newly removed non‐small cell lung cancer (NSCLC) tissue from patients by surgery at the University Hospital in Northern Norway (UNN), as described earlier.^8^ Tumor tissue from nine random NSCLC‐donors was used for CAF‐isolation, and donor characteristics are included in Table 1. CAF cultures have been characterized by the appearance of lineage specific markers; anti‐human FAP (Vitatex) and anti‐human smooth muscle actin (α‐SMA) (Abcam). Isolated CAFs were expanded and cultured in DMEM high glucose basal medium (Sigma Life Science, #D5796) completed with 100 U/mL penicillin and 100 μg/mL streptomycin in addition to 10% Fetal Bovine Serum (FBS). Cells were utilized for investigation at passages 3–6. Normal skin fibroblasts (NSF) used in the presented study were purchased from Evercyte GmbH (Vienna, Austria; # fHDF/TERT166), and maintained and expanded in Gibco® Opti‐MEM™ reduced serum medium (Grand Island, USA, # 31985‐070) supplemented with 100 U/mL penicillin, and 100 mg/mL streptomycin, in addition to 5% FBS.

**TABLE 1:** Cancer‐associated fibroblasts donors information.

Donor number	Age	Sex	Tumor type	T‐size (mm)	T‐stage and N‐stage
1	74	F	Adenosquamous carcinoma	60	pT3N0Mx
2	65	M	Squamous cell carcinoma	24	pT1cN0Mx
3	81	M	Pleomorphic adenocarcinoma	46	pT2bN0Mx
4	68	M	Keratinised squamous cell carcinoma	25	pT1cN0Mx
5	84	M	Adenocarcinoma	75	pT4N0Mx
6	75	M	Squamous cell carcinoma	34	pT2aN0Mx
7	64	F	pT2aN0Mx	12	pT1bN0Mx
8	65	F	Keratinised squamous cell carcinoma	42	pT2bN0Mx
9	72	M	Non‐keratinised squamous cell carcinoma	24	pT2N2Mx

### 2.2 Cell irradiation and treatments

Adherent CAFs were cultivated in T‐75 cell culture flasks and plated in 6‐well plates (5 × 10^5^ cells/well, 80% confluency) one day prior to irradiation with high energy (MV) photons, delivered by a clinical Varian linear accelerator, as described earlier.^8^ Ionizing radiation was provided as one single‐high 18 Gy dose or divided into (3 × 6 Gy) fractionated regimens, using the standard delivery parameters of 30 mm depth, beam quality 15 MV, 6 Gy/min dose‐rate and field size of 20 × 20 cm. Irradiated cells and controls were further incubated (at 37°C) for indicated timepoints and thereafter collected for analyses. In this study, irradiated CAFs are abbreviated as IR‐CAFs.

### 2.3 Western blots

Radioimmunoprecipitation assay (RIPA) buffer (Cell Signaling, Boston, MA, USA) supplied with complete phosphatase and protease inhibitor cocktail (Thermo Fisher Scientific, #78440) was utilized to prepare whole‐cell extracts. Cells were lysed 48 h post‐irradiation. Total cell proteins were separated by NuPAGE™ Bis‐Tris GELS 10% (#NP0316BOX) and transferred onto a PVDF membrane (Invitrogen™, #LC2005). The membranes were then blocked with SuperBlock™ (TBS) blocking buffer (Thermo Fischer Scientific, # 37535) for 2 h at room temperature, and then incubated overnight at 4°C with primary antibodies (Table 2) diluted with Phosphate Buffered saline (PBS)‐T (PBS with 0.5% Tween‐20) supplemented with 0.5% bovine serum albumin (BSA), according to the manufacturers' instructions. Thereafter, membranes were washed (5×) in PBS‐T and then incubated with an anti‐rabbit, anti‐sheep/goat or anti‐mouse Horseradish peroxydase (HRP)‐conjugated secondary antibody (diluted according to the manufacturers' instructions; Table 3) for 1 h at room temperature. In the end, proteins transferred to the membranes were envisioned by enhanced chemiluminescence using an ImageQuant LAS 4000 CCD instrument (GE Healthcare Bio‐Sciences, PA, USA). Relative intensity was evaluated by using the software ImageJ. All western blot images are provided in the supplementary figure for supporting information.

**TABLE 2:** List of primary antibodies used for western blot and immunocytochemistry.

Name	Supplier	Catalog number	Dilution factor for western blot
Human SMAD1/5/8/9	Novus Biologicals	NB100‐56656	1:1000
Human pSMAD1/5/9	Cell Signaling Technology	13820	1:1000
Human SMAD2/3 (C‐8)	Santa Cruz	sc‐133098	1:200
Human pSMAD2/3	Cell Signaling Technology	8828s	1:1000
Human pPDGFR‐β	Cell Signaling Technology	3161	1:500
Human PDGFR‐β	Cell Signaling Technology	3169	1:1000
Human TGF‐β RII	R&D system	AF‐241‐NA	1:100
Human PDGFR‐α (D13C6)	Cell Signaling Technology	5241	1:1000
Human GAPDH	Cell Signaling Technology	2118S	1:1000
β‐actin	Cell Signaling Technology	4970s	1:2000

**TABLE 3:** List of secondary antibodies used for western blot and immunocytochemistry.

Name	Supplier	Catalog number	Dilution factor for western blot
Goat anti‐Mouse IgG (H + L) secondary antibody, HRP	Invitrogen	62‐6520	1:2000
Goat Anti‐Rabbit IgG Antibody, (H + L) HRP conjugate	Chemicon®	AP307P	1:2000
Donkey anti Sheep/Goat IgG:HRP	Serotec	STAR88P	1:10000

For the detection of TGFβR, PDGFRβ, and pPDGFRβ, cells/CAFs were irradiated (3 × 6 Gy or 1 × 18 Gy), incubated for 48 h at 37°C in a cell culture incubator, then treated either with 50 mM PDGF‐BB (PeproTech, #100‐14B) for 45 min at 37°C or left untreated. Afterwards, the cells were lysed as described above and 30 μg total protein/well were applied to the gel.

For the detection of SMAD2/3 and pSMAD2/3, cells/CAFs were treated with IR (3 × 6 Gy or 1 × 18 Gy), incubated for 48 h at 37°C in a cell culture incubator, then incubated with either 20 ng/mL TGF‐β (PeproTech, #100‐21) for 45 min at 37°C or left untreated. Thereafter, cells were lysed and 15 μg total protein per well were applied to the gel.

For the detection of SMAD1/5/8/9 and pSMAD1/5/9, cells/CAFs were irradiated (3 × 6 Gy or 1 × 18 Gy), incubated for 48 h at 37°C in a cell culture incubator, then treated either with 20 ng/mL TGF‐β (PeproTech, #100‐21), or 50 ng/mL BMP2 (PeproTech, #120‐02) for 45 min at 37°C, or left untreated. Cells were subsequently lysed and 15 μg total protein/well was applied to the gel.

### 2.4 Flow cytometry analyses

Surface expression of TGFβRII and PDGFRβ on control and irradiated CAFs (IR‐CAFs) was examined by flow cytometry on BD FACSAria III instrument, and by the FlowJo software, Ver.7.2.4 (Tree Star‐USA). In short, CAF/IR‐CAF cells (2.5 × 10^5^ cells/condition) were immuno‐labeled (30 min at 4°C) with several fluorescent antibodies specific for each distinct surface marker (Table 4). Isotype controls included REA control (#130‐104‐612; Miltenyi Biotec), mouse IgG1 (#130‐098‐845; Miltenyi Biotec), and mouse IgG2a (#555574; BD Biosciences‐USA).

**TABLE 4:** List of antibodies used for flow cytometry.

Name	Supplier	Catalog number
TGF‐β RII Antibody, anti‐human, FITC, REAfinity™	Miltenyi Biotec	130‐115‐065
PDGFR‐α Antibody, anti‐human, APC, REAfinity™	Miltenyi Biotec	130‐115‐338
PDGFR‐β Antibody, anti‐human, PE‐Vio® 770, REAfinity™	Miltenyi Biotec	130‐105‐323

### 2.5 ELISA

Quantitative protein determinations of TGF‐β and PDGF‐BB in culture supernatants from IR‐CAFs/CAFs were performed by using Enzyme‐link Immunosorbent Assay (ELISA) kits (R&D Systems, Minneapolis, MN, USA, # DY240‐05 and # DY220), as stated in the manufacturer's instructions. Briefly, conditioned medium (CM), supplied with 10% FBS from IR‐CAFs/CAFs was collected from cultures incubated for 48 h post‐irradiation, centrifuged at 2000× *g* (4°C, 10 min), filtrated with pore size 0.45 μm and stored at −80 °C before ELISA analyses. Absorbance at 450 nm for each sample was measured by SpectraMax Plus 384 Microplate Reader (Molecular Devices, CA, USA).

### 2.6 Immunocytochemistry

CAFs in suspension were seeded in 4‐well chamber slides (1 × 10^4^ cells/well) (Nunc, Thermo Fisher Scientific, NY, USA), incubated (37°C) in standard CAF culture medium for 24 h, and then irradiated. At the indicated timepoints, cells were fixed with 4% PFA/PBS for 10 min and then permeabilized with 0.2% Triton‐PBS for 8 min. Upon removing the permeabilizing solution, the slides were treated with blocking buffer (2% HSA‐PBS). Subsequently, primary antibodies (Table 2) were diluted in blocking buffer, then incubated with CAFs for 45 min at room temperature. Next, cells were washed and incubated with secondary antibodies (Table 3) in blocking buffer for 30 min at 20°C, and thereafter washed and mounted in DAPI‐FluoromountG mounting solution (#0100‐20, Southern Biotech, Birmingham, AL, USA). Specimens were evaluated in a fluorescence microscope (Zeiss Axiophot, Germany) mounted with a Nikon DS‐5MC digital camera, and images were prepared with an Adobe® Photoshop Software (CS5).

### 2.7 Statistics

All statistical analyses were carried out by using the GraphPad Prism software (GraphPad Software, Inc, La Jolla, CA). Flow cytometry data between treatment groups were compared and analyzed by employing the Brown‐Forsythe and Welch ANOVA test. Significance values were corrected by Dunnett's T3 correction for multiple comparisons. The western blot experimental results were examined by utilizing either one‐way ANOVA test (significance values corrected by Dunnett correction for multiple comparisons), or Kruskal–Wallis test with significance values corrected by Dunn correction for multiple comparisons where requirement of Gaussian distribution of residuals was not met. *p* < .05 indicates chosen significance level. The results were exhibited in graphs where each individual dot represents each donor in the dataset. Regarding ELISA experiments, only read‐outs above the assay detecting limit are shown.

## 3 RESULTS

### 3.1 Radiation effects on expression of TGF‐β receptor and ligand in CAFs

**FIGURE 1:**
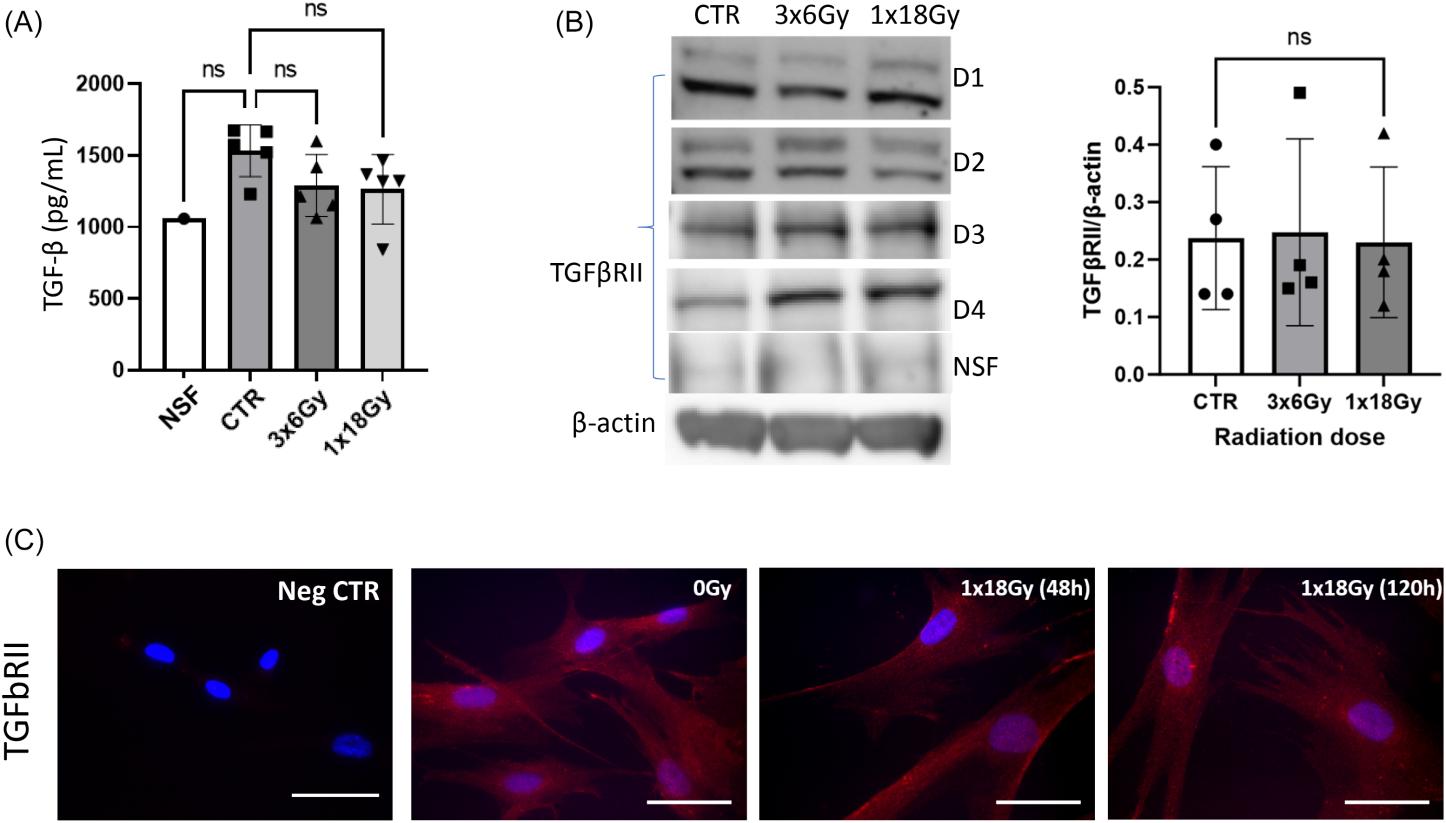
Radiation effects on the expression of TGFβRII (receptor) and soluble TGF‐β (ligand) in cancer‐associated fibroblasts (CAFs). (A) TGF‐β release in culture supernatants measured by ELISA; (B) TGFβRII receptor expression in CAF cell lysates analyzed by western blot. Statistics were done by Kruskal–Wallis test with significance values corrected by Dunn correction for multiple comparisons. In A and B, Control (CTR) refers to nonirradiated CAFs, and data represents mean (± SD) values from four different CAF donors. Loading controls with β‐actin in B (for TGFβRII) and Figure 2B (for PDGFR‐β) correspond to the same CAF donor (donor 3). (C) Immunostaining of irradiated and nonirradiated CAFs, using specific anti‐TGFβRII antibody (red) and nuclear DAPI (blue). NSF refers to normal skin fibroblast.

Initial experiments were aimed at exploring potential changes provoked by IR directly on the protein expression‐levels of TGF‐β and/or TGF‐β receptor (TGFβRII) in CAFs. Secreted levels of TGF‐β from CAFs and IR‐CAFs were measured in culture media conditioned for 48 h, between day 3 and day 5 post‐irradiation. Results indicated that CAFs secrete higher amounts of TGF‐β than normal lung fibroblasts (nearly significant differences), however, radiation exposure was unable to exert measurable changes in the release of TGF‐β by CAFs (Figure 1A). Likewise, TGF‐β receptor expression was compared between IR‐CAFs and control CAFs by different approaches. Results from western blots indicated that TGFβRII expression in whole cell lysates remain unchanged following radiation at both single‐high dose and fractionated regimens (Figure 1B). Same outcomes were seen by immunofluorescence staining and imaging at different time points post‐radiation (Figure 1C).

### 3.2 Radiation effects on expression of PDGF receptors and ligand in CAFs

**FIGURE 2:**
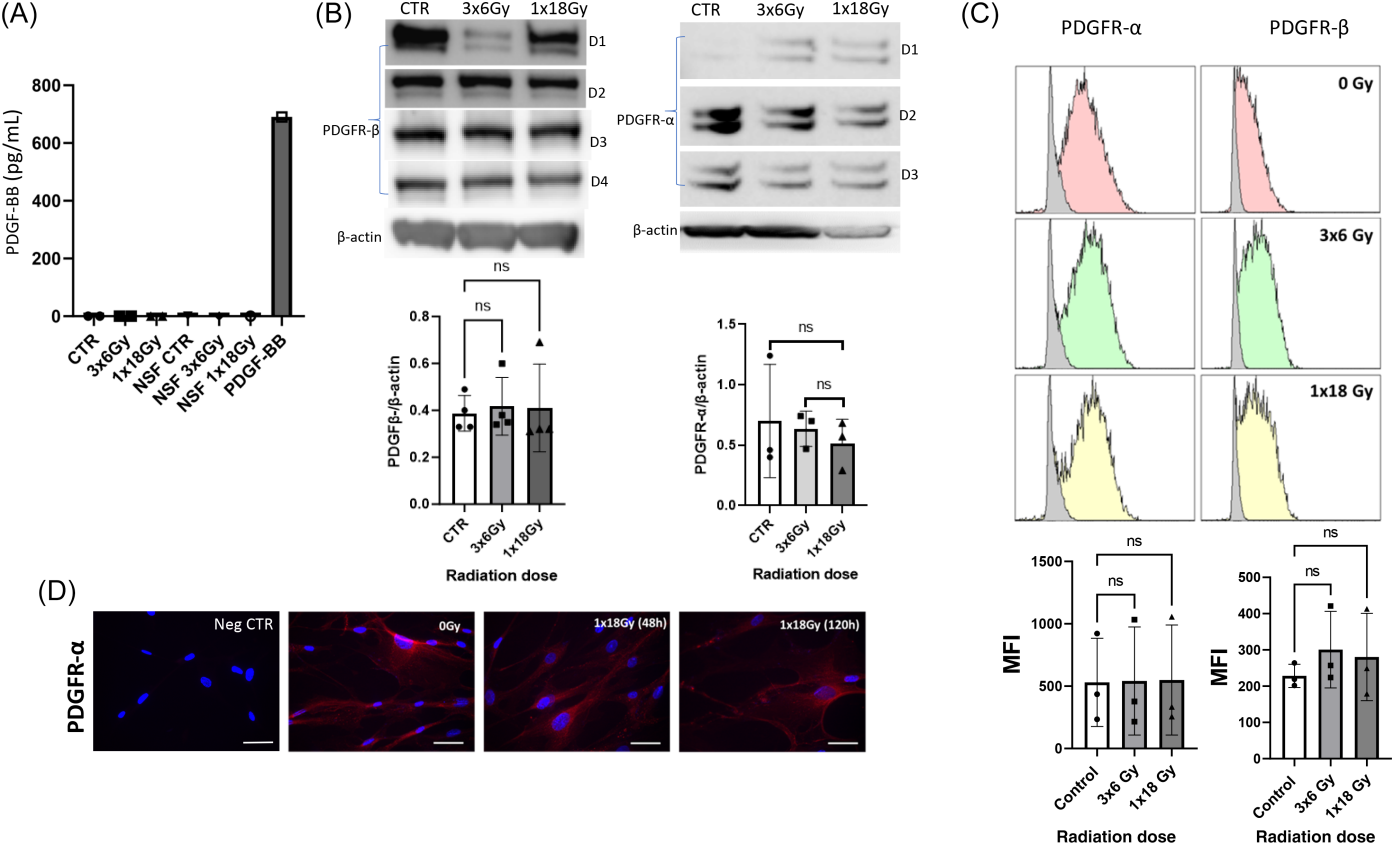
Radiation effects on the expression of PDGFRα/β (receptors) and PDGF‐BB (ligand) in cancer‐associated fibroblasts (CAFs). (A) PDGF‐BB protein level in culture supernatants measured by ELISA. Recombinant human PDGF‐BB is used as positive control; (B) PDGFR‐α and PDGFR‐β receptor expression in CAF cell lysates analyzed by western blot. Statistics were done by Kruskal–Wallis test with significance values corrected by Dunn correction for multiple comparisons. Loading controls with β‐actin in Figure 1B (for TGFβRII) and B (for PDGFRβ) correspond to the same CAF donor (donor 3). (C) Surface expression of PDGFR‐α/β receptors on CAF measured by flow cytometry. Statistics were done by Brown–Forsythe and Welch ANOVA test. Data represents mean (± SD) values from three unrelated CAF donors in A and C, and from three or four donors in B; and (D) Immunostaining of irradiated and nonirradiated CAFs, using specific anti‐PDGFR‐α antibody (red) and nuclear DAPI (blue).

In parallel, we also investigated potential changes induced by irradiation on the expression of PDGF ligand and receptors. Our ELISA results indicate that neither CAFs nor NFs express measurable levels of PDGF‐BB at any of the tested conditions, including irradiated cultures (Figure 2A). In parallel, we measured expression of PDGFRα/β in CAFs from four unrelated, random donors in irradiated and nonirradiated conditions. As for the case of TGFβRII, expression of PDGFRβ in CAFs (measured in whole cell lysates) by western blot was unaffected after irradiation for both regimens (Figure 2B). Outcomes from flow cytometry analyses were in line with observations by western blots, confirming that expression of PDGFR‐α/β in CAFs is unaffected by irradiation (Figure 2C). By immunofluorescence, we could observe homogenous expression of PDGFR‐α at the cell surface, and no apparent changes in receptor expression after irradiation (Figure 2D). Unfortunately, the PDGFR‐β antibody used in our study was not well suited for doing immunocytochemistry assays (data not shown).

### 3.3 Radiation effects on TGF‐β and PDGF receptor signaling

**FIGURE 3:**
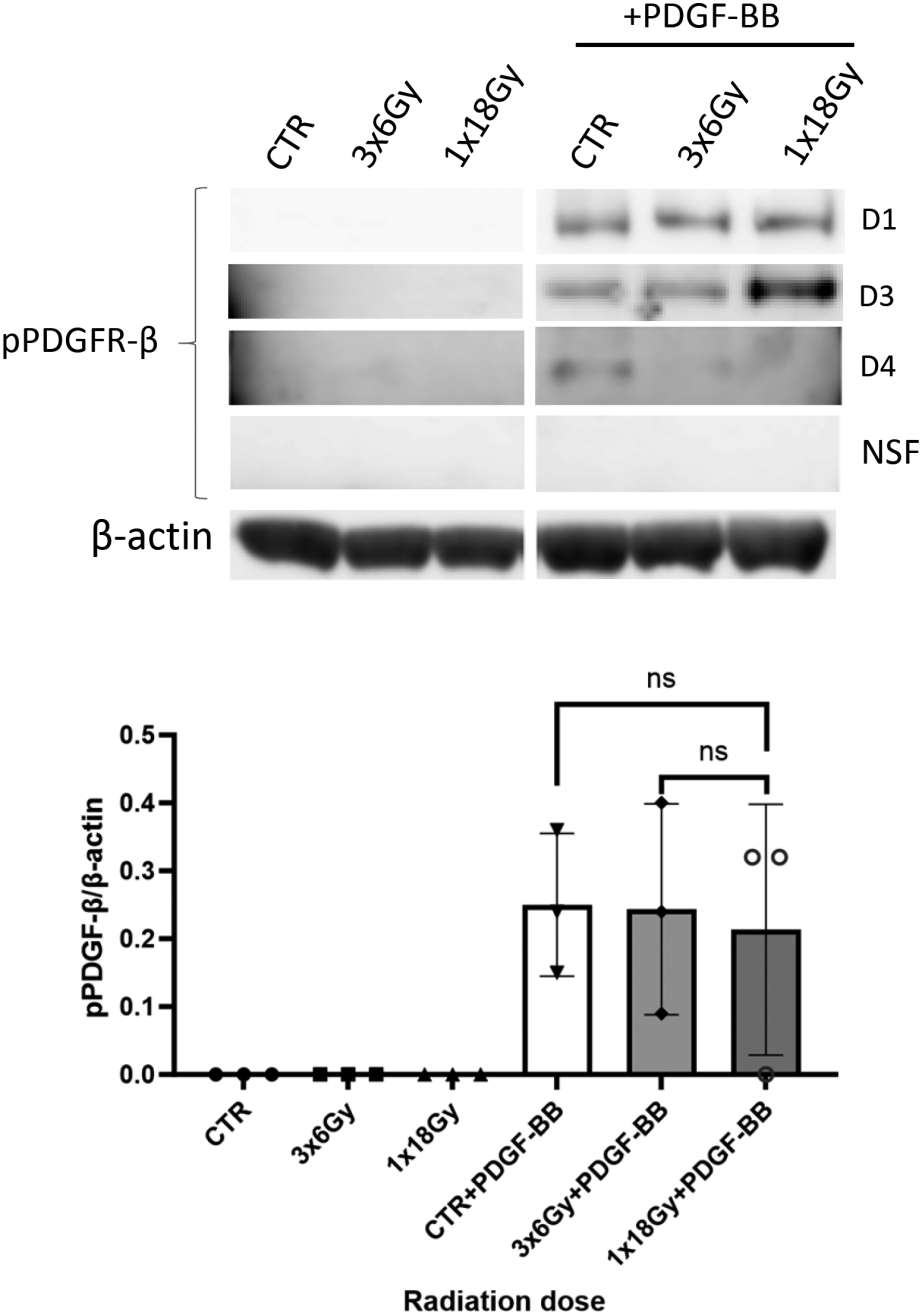
Radiation‐effects of PDGFR‐β receptor signaling. PDGFR‐β phosphorylation in the absence or presence of PDGF‐BB was evaluated in irradiated and control cancer‐associated fibroblasts (CAFs) by western blot. Data represents mean (± SD) values from three different CAF donors. Statistical *p*‐values were determined using ordinary one‐way ANOVA with Dunnett correction for multiple comparisons.

**FIGURE 4:**
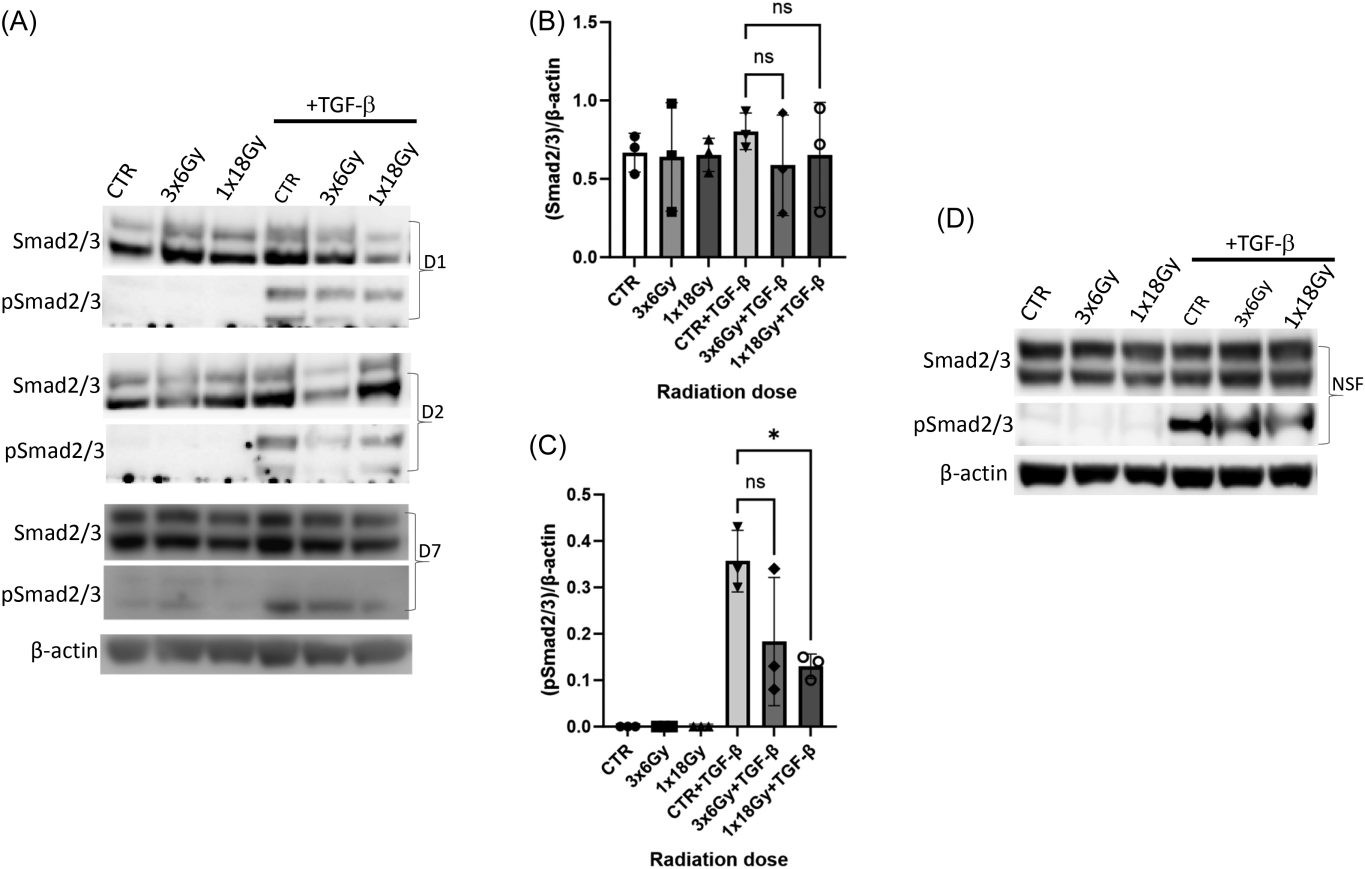
Radiation effects on TGF‐β receptor signaling via canonical pathway. (A) Smad2/3 and pSmad2/3 expression in irradiated and control cancer‐associated fibroblasts (CAF) treated or not with TFG‐β1 measured in cell lysates by western blot. (B) Quantitative analyses of Smad2/3 expression in cell samples. (C) Quantitative analyses of pSmad2/3 in cell samples. In B and C, data represents mean (± SD) values from three different CAF donors. Statistical *p*‐values were determined using ordinary one‐way ANOVA with Dunnett correction for multiple comparisons. (D) Smad2/3 and pSmad2/3 expression in normal skin fibroblasts (NSF) cell lysates analyzed by western blot.

**FIGURE 5:**
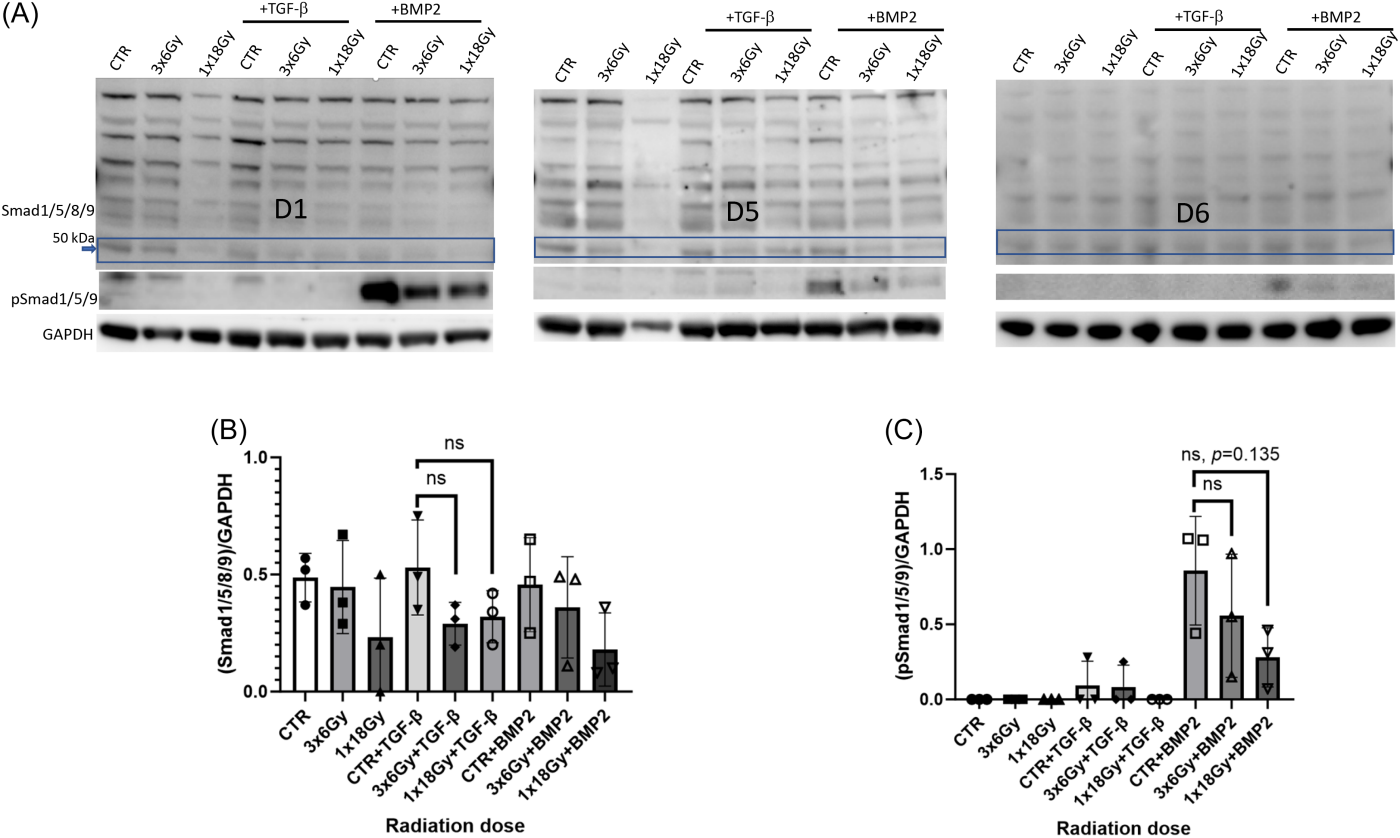
Radiation effects on TGF‐β receptor signaling via noncanonical pathway. (A) Smad1/5/8/9 and pSmad1/5/9 expression in cancer‐associated fibroblasts (CAF) cell lysates analyzed by western blot. (B) quantitative analyses of Smad1/5/8/9 expression in cell samples; (C) quantitative analyses of pSmad1/5/9 expression in cell samples. In B and C, data for pSmad1/5/9 represents mean (± SD) values from three different CAF donors. Statistical *p*‐values between CTR (nonirradiated) and irradiated CAFs were determined using ordinary one‐way ANOVA with Dunnett correction for multiple comparisons.

Next, we explored potential effects of irradiation on TGF‐β and PDGF receptor signaling. PDGFR‐β activation was measured by means of receptor phosphorylation, whereas TGFβRII activation was determined by detection of Smad2/3 phosphorylation for the canonical pathway, or Smad 1/5/8 phosphorylation for the alternative pathway. In the absence of exogenously added ligands, TGF‐β or PDGF receptor activation was not observed in either irradiated or control groups (Figures 3 and 4). Upon stimulation of cells with cognate ligands (recombinant human rhPDGF‐BB and rhTGF‐β1), we could register PDGFR‐β and Smad 2/3 phosphorylation, respectively (Figures 3 and 4A–C). Evidently, PDGFR‐β phosphorylation in the presence of PDGF‐BB was unchanged in CAFs exposed to radiation (Figure 3). However, signaling via pSmad2/3 was down‐regulated in (1 × 18 Gy) IR‐CAFs upon stimulation with TGF‐β1 when compared with controls (Figure 4A,C), Notably, receptor signaling via the alternative pathway (pSmad1/5/8) was only activated in the presence of bone morphogenic protein‐2 (BMP2) and not TGF‐β (Figure 5A–C), and this signaling was also moderately down‐regulated in IR‐CAFs irradiated with a single‐high dose but without reaching significance (Figure 5A–C).

## 4 DISCUSSION

The role played by CAFs on radiotherapy resistance remains controversial, and the effects that irradiation exerts on CAFs are still incompletely understood. In this study, we explore potential effects exerted by radiation on the TGF‐β/TGFβR and PDGF/PDGFR signaling system in CAFs. Results from this work reveal that radiation does not exert measurable effects on the protein expression of these two receptor‐ligand systems in CAFs. Likewise, we demonstrate that radiation per se is unable to trigger TGFβR or PDGFR‐β receptor activation. Interestingly, radiation given at a high dose of 18 Gy had the tendency to decrease both Smad2/3‐dependent and Smad1/5/8‐dependent TGFβR signaling in the presence of exogenously added TGF‐β and BMP2, respectively, although for the case of the noncanonical pathway the values did not reach statistical significance due to substantial differences between CAF donors.

PDGF is considered to act primarily in a paracrine fashion in epithelial tumors, influencing stromal cells for instance fibroblasts, mesenchymal cells, and pericytes.^20^ In experimental models, ligand‐mediated activation of PDGFR signaling triggers fibroblasts recruitment, proliferation, and differentiation. In CAFs, PDGFR signaling may, in addition, affect ECM sediment and tissue stiffness.^52^ In agreement with the described effects, higher expression of stromal PDGFR‐β has been correlated with poor prognosis in various cancer types.^19^, ^53^, ^54^ Moreover, the PDGF/PDGFR route has been crucial in the regulation of tumor angiogenesis and lymphangiogenesis. The significance of PDGFR‐β‐positive perivascular cells in tumor vessel stabilization was documented in quite some studies.^21^

In the context of radiation or radiotherapy, there are few or no published records on the effects of radiation on the expression of PDGF and/or PDGFRs in stromal cells. In a recent study by Strell et al. performed on mammary ductal carcinoma in situ (DCIS) patient samples, authors found an association between high tissue expression of PDGFR‐β and reduced RT sensitivity, indicating a potential role of PDGFR‐β positive cells in radiotherapy resistance.^55^ Results from our study indicate that exposure of CAFs to intermediate or high radiation doses does not have a significant impact on PDGFR‐β expression or signaling in CAFs, suggesting that radiation exposure does not trigger PDGFR‐mediated tumor radioresistance. To increase the biological relevance of the results generated in our study, we have used CAF cultures from different patients in experiments. Although the combined results showed no statistically significant variations in PDGFR‐β expression or phosphorylation after radiation, in some experiments we observe different responses between donors, indicating that some changes can still take place in a donor‐dependent manner.

The effects of active TGF‐β in cancer development are multifactorial and context‐dependent. As one of the lead elements of the TME, CAFs are both a source and a target of TGF‐β. In experimental breast cancer models, TGF‐β and SDF‐1 form part of two autocrine and cross‐linking signaling loops that push CAF/myofibroblast extension at the invasive front.^56^, ^57^ CAF‐secreted TGF‐β and SDF‐1 also participate in the promotion of EMT and enhance proliferation of cancer cells.^57^, ^58^, ^59^ Because of the TGF‐β‐mediated myofibroblastic trans‐differentiation, ECM secretion by TGF‐β‐stimulated CAFs is increased, thus contributing to enhanced tumor density and matrix stiffness. Moreover, TGF‐β‐mediated ECM remodeling has a direct impact on tumor cell migration, immune cell infiltration, vascularization, drug delivery in addition to integrin‐mediated signaling.^60^

Previous reports have highlighted the importance of TGF‐β on the CAF‐tumor cell interplay in connection to radiotherapy. In an early investigation employing oral squamous cell carcinoma cells and fibroblasts cell lines, irradiated fibroblasts promoted invasion and expansion of SCC cells by augmenting invasive growth‐related molecules levels through TGF‐β1‐mediated bystander mechanisms.^61^ It has also been proposed that TGF‐β from pancreatic stellate cells participate in tumor progression and radioresistance by promoting EMT and stem cells phenotypes in pancreatic cancer cells.^62^ On the contrary, in the study by Arshad et al. authors observed that irradiation of fibroblasts and murine lung tumor cells in cocultures represses the expression of TGF‐β, MMPs, and other soluble factors, thus abolishing the pro‐migratory phenotype of cancer cells.^9^ To our knowledge, the effects of radiation on TGF‐β receptor and/or signaling in CAFs have not been reported previously. Our data indicate that radiation is not able to trigger measurable changes in expression of TGF‐β receptor (TGFβRII) or ligand in CAFs, however, TGF‐β receptor signaling seems to be attenuated in IR‐CAFs. Consequently, radiation could exert beneficial effects in the TME by ameliorating the negative outcomes associated to TGFβ‐mediated activation of CAFs and the downstream effects of such activation. In this regard, early studies by our laboratory showed that the proliferative and migratory properties of lung tumor cell lines exposed to CM from irradiated and nonirradiated lung CAFs were indeed unaffected.^63^ Interestingly, the pro‐tumorigenic effects exerted by CAFs in tumor growth upon transplantation in mice were lost when tumor cells were co‐transplanted with irradiated CAFs.^10^ Results from this study might partially explain the loss of pro‐tumorigenic functions exerted by CAFs.

In the present study, we have explored the potential effects of radiation on the expression of TGF‐β and PDGF receptors. For this purpose, we have used a multimodality approach, including flow cytometry, western blots, and immunofluorescence. A strength of our experimental model is the use of freshly isolated CAFs from tumor specimens collected from multiple independent donors. Radiation treatments in this study included two different regimens, comprising a single high dose regimen and a fractionated regimen, however, radiation effects have been measured at a single time point, normally 5 days after treatment, when a stable senescent phenotype is acquired. In our study, we have not included radiation effects on normal fibroblasts which could have given insights into possible normal tissue reactions to radiotherapy. Moreover, in this study we do not have data from coculture conditions with other cell types. Cellular crosstalk in the context of tumors is important, and the presence of tumor cells or other cells from the TME could have had an influence in the outcomes of the study. Hence, outcomes from our study should also be confirmed in coculture settings and in in vivo models, and ideally in human specimens in cancer types when the treatment protocols allow for collection of tumor samples before and after therapy.

## 5 CONCLUSION

This is the first study ever reporting on the effects of radiation on the expression and signaling patterns of TGF‐β and PDGF receptors in CAFs. Results from this in vitro study indicate that exposure of CAFs to irradiation does not have a measurable impact on the expression of PDGF and/or TGF‐β receptors and ligands. Likewise, radiation exposure is not able for itself to induce activation of these receptors. However, a reduction in TGFβR signaling is observed in high‐dose IR‐CAFs, which in turn it could contribute to a reduction in TGF‐β‐mediated CAF activation and their associated tumor‐promoting effects. In clinical settings, therapeutic strategies that comprise inhibition of TGFβR and/or PDGFR‐mediated signaling in CAFs could be considered to abrogate or ameliorate stroma‐derived therapy resistant mechanisms, and radiotherapy can be safely applied in the treatment plan since, as shown in this study, it would have synergistic effects.

## AUTHOR CONTRIBUTIONS

**Nannan Yang:** Formal analysis (lead); investigation (lead); methodology (lead); validation (lead); visualization (lead); writing – review and editing (equal). **Turid Hellevik:** Conceptualization (equal); writing – review and editing (equal). **Rodrigo Berzaghi:** Formal analysis (supporting); investigation (supporting); methodology (supporting); writing – review and editing (supporting). **Inigo Martinez‐Zubiaurre:** Conceptualization (equal); project administration (lead); supervision (lead); writing – original draft (lead).

## FUNDING INFORMATION

This work was supported by the Department of Clinical Medicine and the Department of Community Medicine, Faculty of Health Sciences, UiT The Arctic University of Norway; by grants from the Norwegian Regional Health Authorities (Grants #: HNF1423‐18 to TH and HNF 1337‐17 to RB), the Aakre Foundation at UiT and The Norwegian Cancer Society (project ID#: 198164). A grant from the publication fund at UiT The Arctic University of Norway is utilized to cover the publication cost of this article.

## CONFLICT OF INTEREST STATEMENT

The authors claim that there are no conflicts of interest connected to this work.

## ETHICS STATEMENT

All methods relating to human material were carried out in compliance with applicable ethical guidelines and regulations. The employment of human material that has been included in this study (REK Nord 2014/401; 2016/714; 2016/2307) has been approved by the Regional Ethical Committee of Northern Norway. All human samples were used anonymously and were collected upon informed consent from patients.

## Supporting information


**Figure S1.** Uncropped scans of western blots found in Figure 1C and 2B, donor 1 and 2. CTR refers to nonirradiated CAFs.
Figure **S2.** Uncropped scans of western blots found in Figure 1C and 2B, donor 3, 4 and NSF.
Figure **S3.** Scans of western blots found in Figure 2B, donor 1–3.Figure **S4.** Uncropped scans of western blots found in Figure 3, donor 1, 3, 4 and NSF.
Figure **S5.** Uncropped scans of western blots found in Figure 4A, donor 1 and 2.
Figure **S6.** Uncropped scans of western blots found in Figure 4A, donor 3, 7 and NSF.
Figure **S7.** Uncropped scans of western blots found in Figure 4, and donor 1 and 2.
Figure **S8.** Uncropped scans of western blots found in Figure 4, donor 3, 7 and NSF.
Figure **S9.** NSF western blot result for Smad1/5/8/9 and pSmad1/5/9.

## Data Availability

Data is available from the corresponding author upon request.
