# Supplementary material for: Radiation‐induced effects on TGF‐β and PDGF receptor signaling in cancer‐associated fibroblasts
Source: Cancer Rep (Hoboken). 2024 Mar 15;7(3):e2018. doi: 10.1002/cnr2.2018 (PMC10941573; doi:10.1002/cnr2.2018)
Supplement: Supplementary file 1 — Figure S1. Uncropped scans of western blots found in Figure 1C and 2B, donor 1 and 2. CTR refers to nonirradiated CAFs. Figure S2. Uncropped scans of western blots found in Figure 1C and 2B, donor 3, 4 and NSF. Figure S3. Scans of western blots found in Figure 2B, donor 1–3.Figure S4. Uncropped scans of western blots found in Figure 3, donor 1, 3, 4 and NSF. Figure S5. Uncropped scans of western blots found in Figure 4A, donor 1 and 2. Figure S6. Uncropped scans of western blots found in Figure 4A, donor 3, 7 and NSF. Figure S7. Uncropped scans of western blots found in Figure 4, and donor 1 and 2. Figure S8. Uncropped scans of western blots found in Figure 4, donor 3, 7 and NSF. Figure S9. NSF western blot result for Smad1/5/8/9 and pSmad1/5/9. [file CNR2-7-e2018-s001.pptx]

## Slide 1
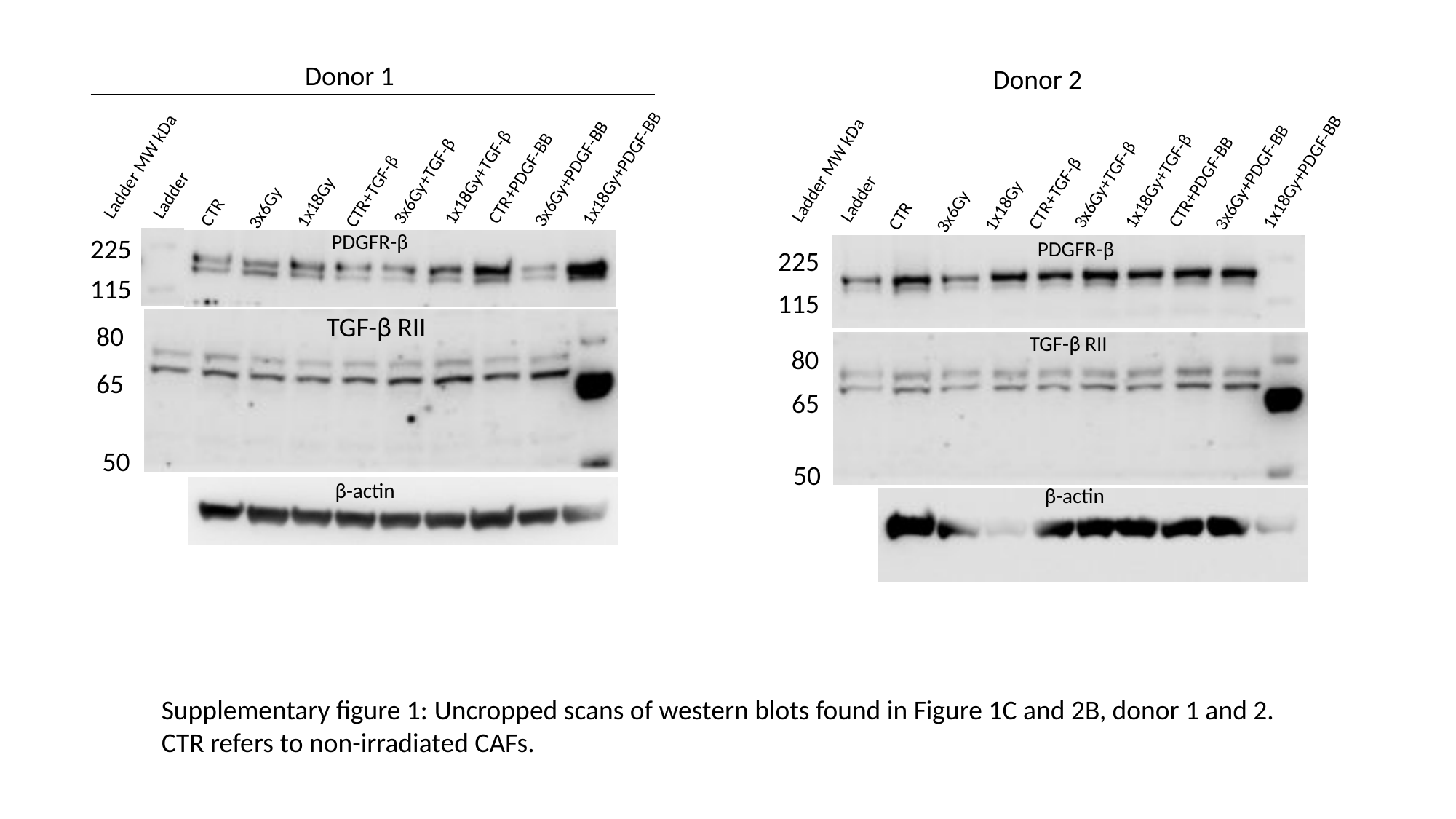

Donor 1
Donor 2
Ladder MW kDa
Ladder
Ladder MW kDa
Ladder
 1x18Gy+PDGF-BB
CTR
 1x18Gy+PDGF-BB
1x18Gy
3x6Gy
CTR
 1x18Gy+TGF-β
 3x6Gy+PDGF-BB
 CTR+TGF-β
1x18Gy
 CTR+PDGF-BB
 3x6Gy+TGF-β
3x6Gy
 CTR+TGF-β
 1x18Gy+TGF-β
 3x6Gy+PDGF-BB
 CTR+PDGF-BB
 3x6Gy+TGF-β
PDGFR-β
225
PDGFR-β
225
115
115
TGF-β RII
 80
TGF-β RII
 80
 65
 65
 50
 50
β-actin
β-actin
Supplementary figure 1: Uncropped scans of western blots found in Figure 1C and 2B, donor 1 and 2.
CTR refers to non-irradiated CAFs.

## Slide 2
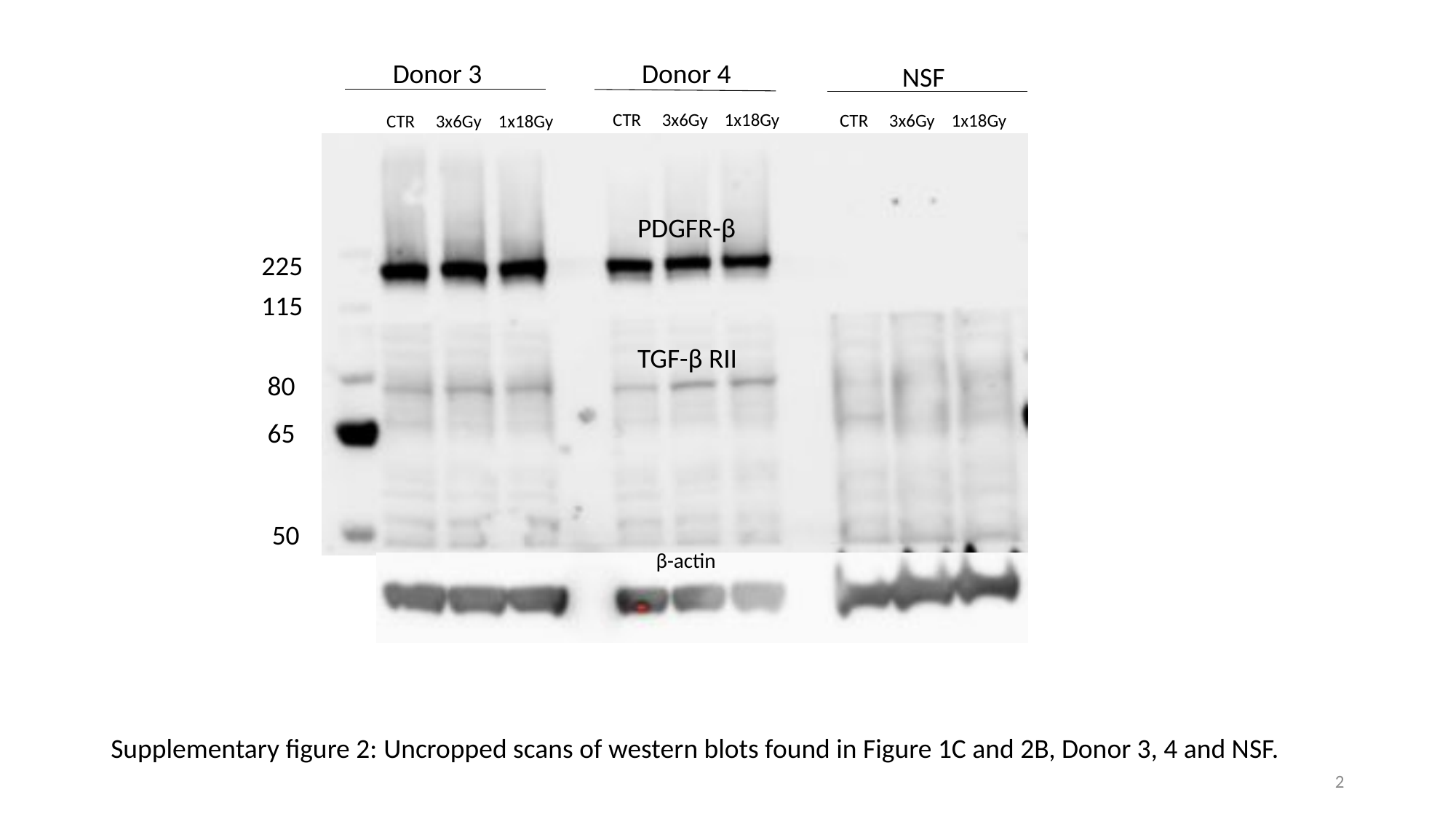

Donor 3
 Donor 4
 NSF
 CTR 3x6Gy 1x18Gy
 CTR 3x6Gy 1x18Gy
 CTR 3x6Gy 1x18Gy
PDGFR-β
225
115
TGF-β RII
 80
 65
 50
β-actin
Supplementary figure 2: Uncropped scans of western blots found in Figure 1C and 2B, Donor 3, 4 and NSF.
2

## Slide 3
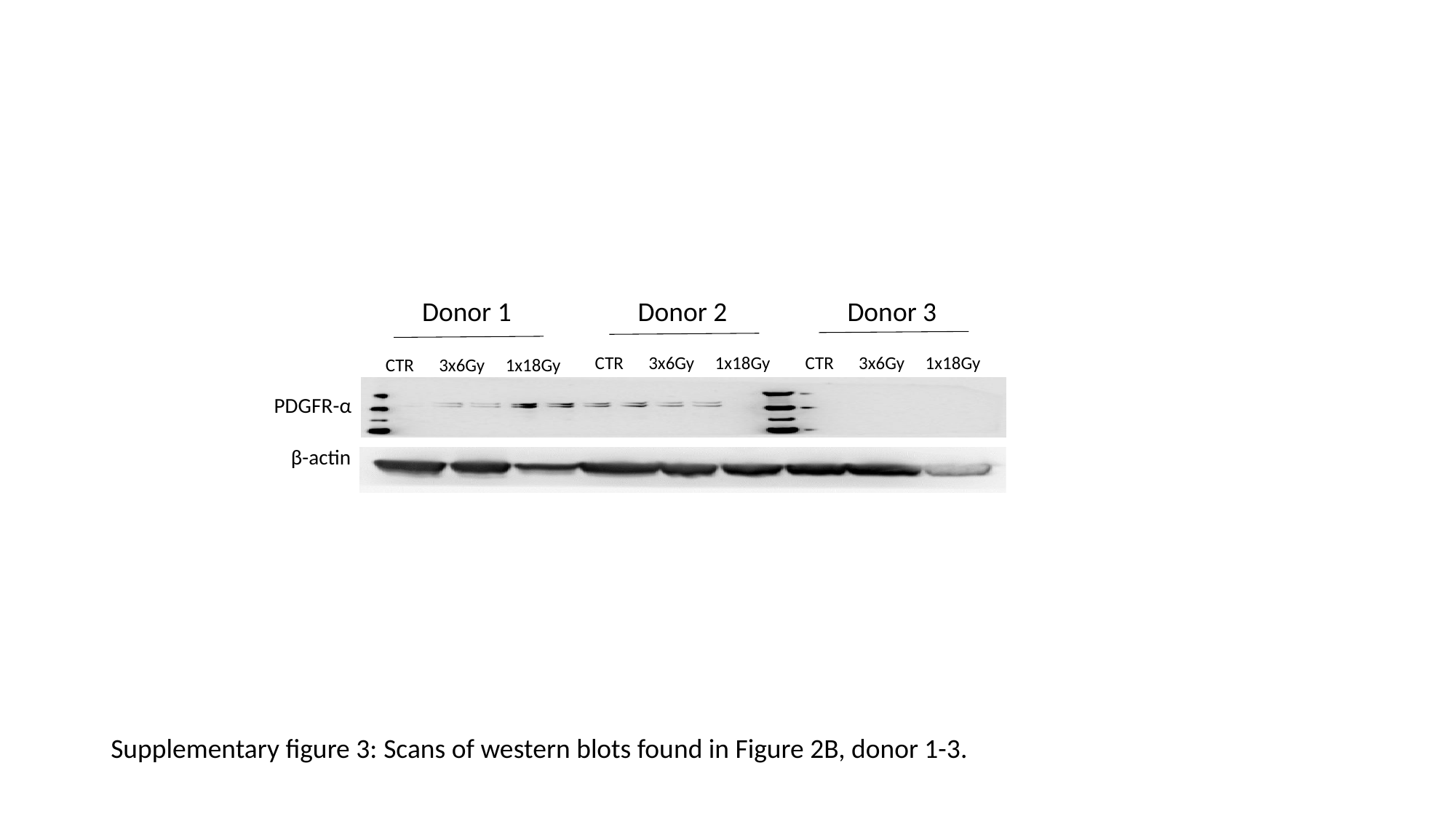

Donor 1
Donor 2
Donor 3
 CTR 3x6Gy 1x18Gy
 CTR 3x6Gy 1x18Gy
 CTR 3x6Gy 1x18Gy
PDGFR-α
β-actin
Supplementary figure 3: Scans of western blots found in Figure 2B, donor 1-3.

## Slide 4
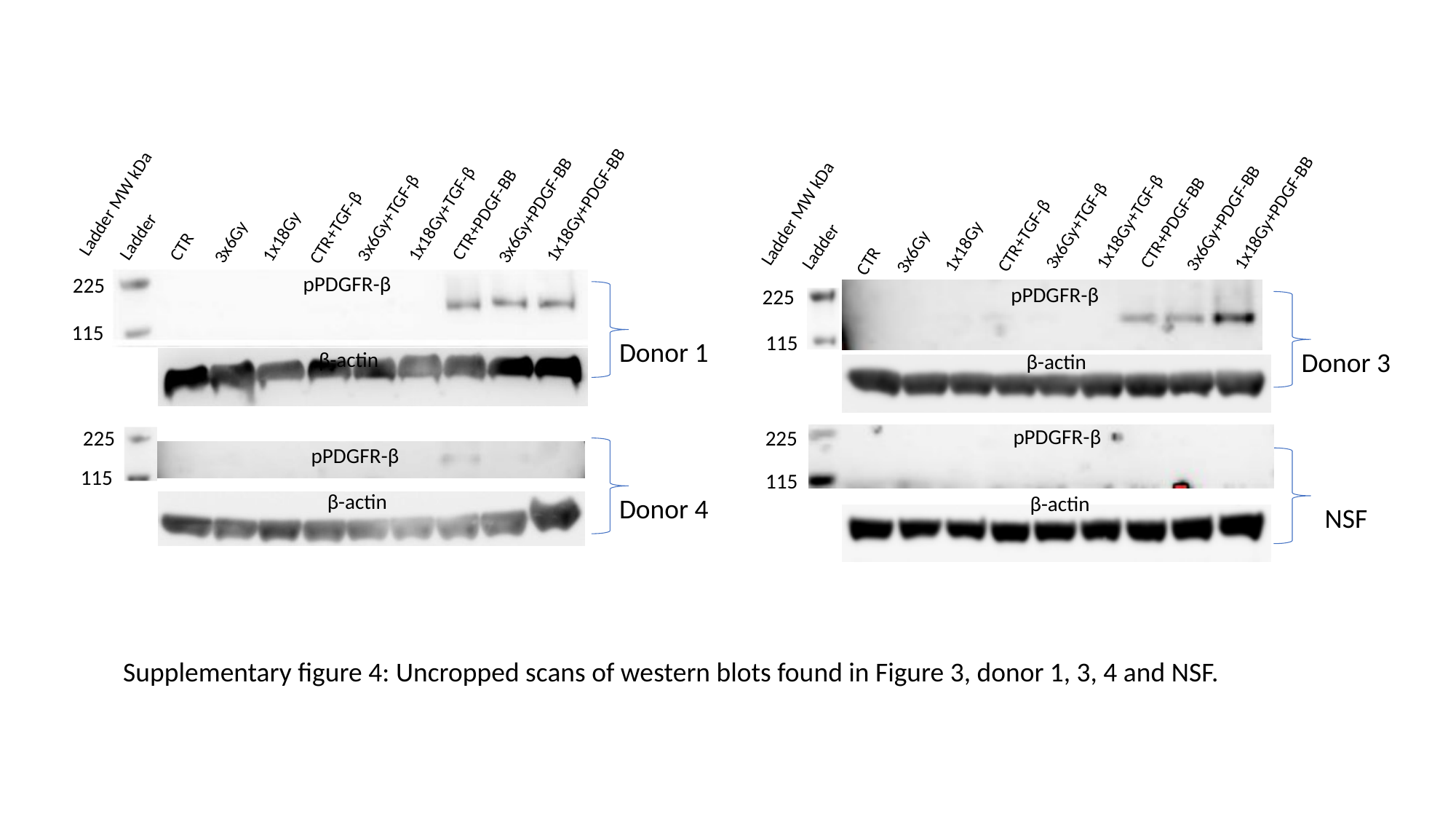

Ladder MW kDa
 1x18Gy+PDGF-BB
CTR
Ladder
1x18Gy
3x6Gy
Ladder MW kDa
 1x18Gy+TGF-β
 3x6Gy+PDGF-BB
 CTR+TGF-β
 CTR+PDGF-BB
 1x18Gy+PDGF-BB
 3x6Gy+TGF-β
Ladder
1x18Gy
3x6Gy
 1x18Gy+TGF-β
 3x6Gy+PDGF-BB
CTR
 CTR+TGF-β
 CTR+PDGF-BB
 3x6Gy+TGF-β
pPDGFR-β
225
225
pPDGFR-β
115
115
Donor 1
Donor 3
β-actin
β-actin
225
225
pPDGFR-β
pPDGFR-β
115
115
β-actin
β-actin
Donor 4
NSF
Supplementary figure 4: Uncropped scans of western blots found in Figure 3, donor 1, 3, 4 and NSF.

## Slide 5
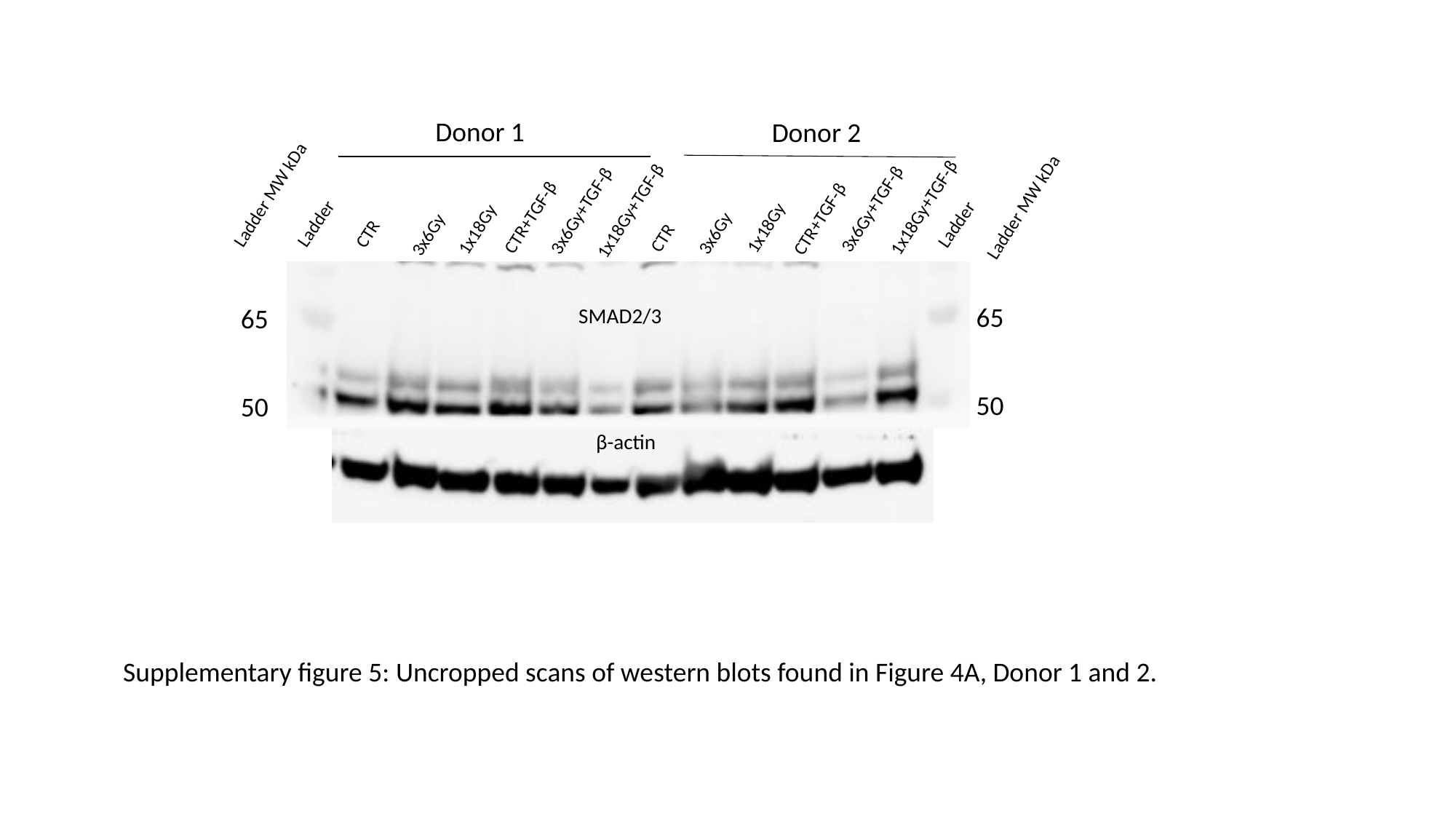

Donor 1
 Donor 2
Ladder MW kDa
CTR
Ladder
Ladder
CTR
1x18Gy
1x18Gy
3x6Gy
3x6Gy
 CTR+TGF-β
Ladder MW kDa
 CTR+TGF-β
 3x6Gy+TGF-β
 1x18Gy+TGF-β
 3x6Gy+TGF-β
 1x18Gy+TGF-β
 65
 65
SMAD2/3
 50
 50
β-actin
Supplementary figure 5: Uncropped scans of western blots found in Figure 4A, Donor 1 and 2.

## Slide 6
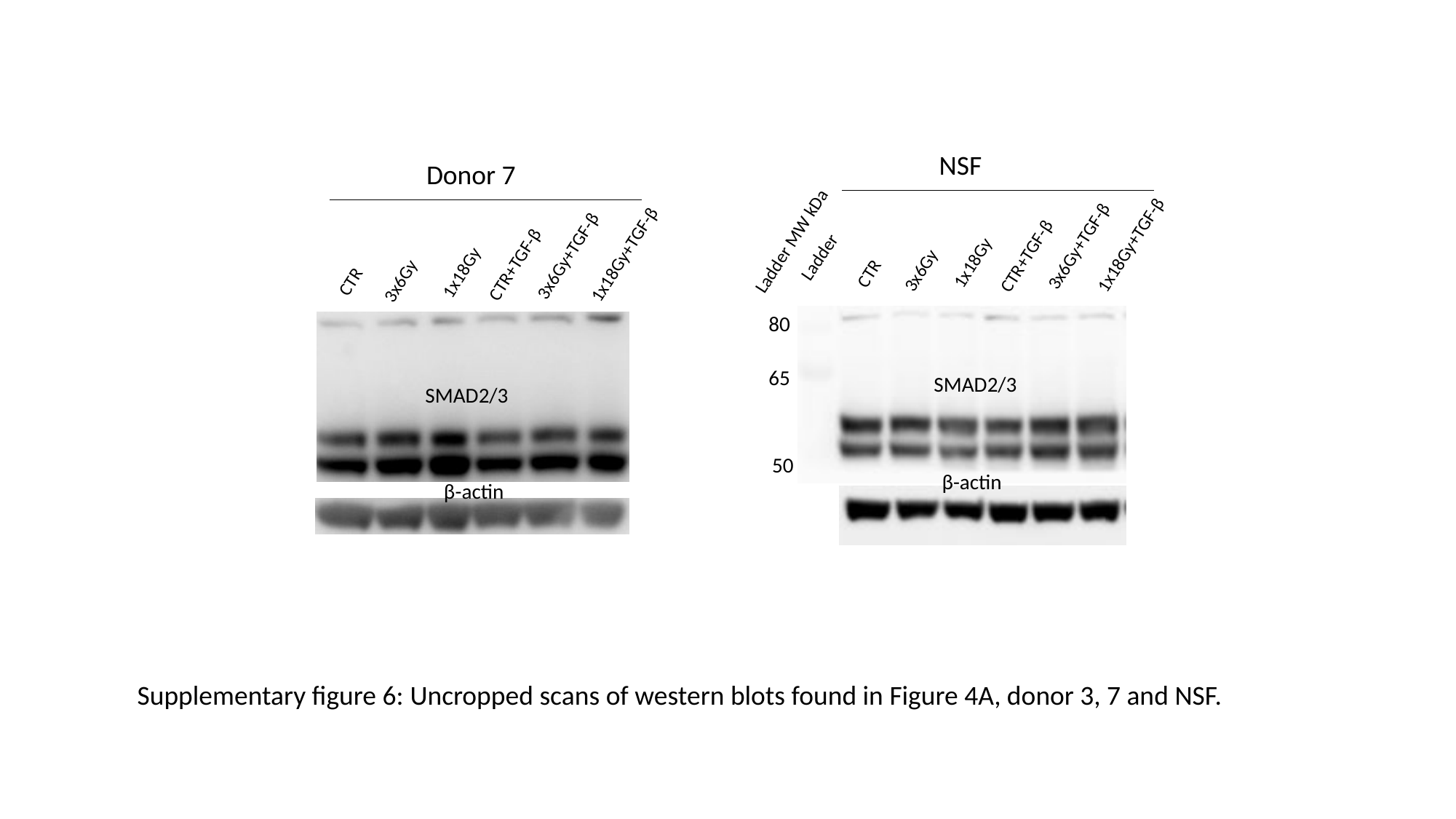

NSF
 Donor 7
Ladder
CTR
1x18Gy
3x6Gy
Ladder MW kDa
CTR
 CTR+TGF-β
 3x6Gy+TGF-β
 1x18Gy+TGF-β
1x18Gy
3x6Gy
 CTR+TGF-β
 3x6Gy+TGF-β
 1x18Gy+TGF-β
 80
 65
SMAD2/3
SMAD2/3
 50
β-actin
β-actin
Supplementary figure 6: Uncropped scans of western blots found in Figure 4A, donor 3, 7 and NSF.

## Slide 7
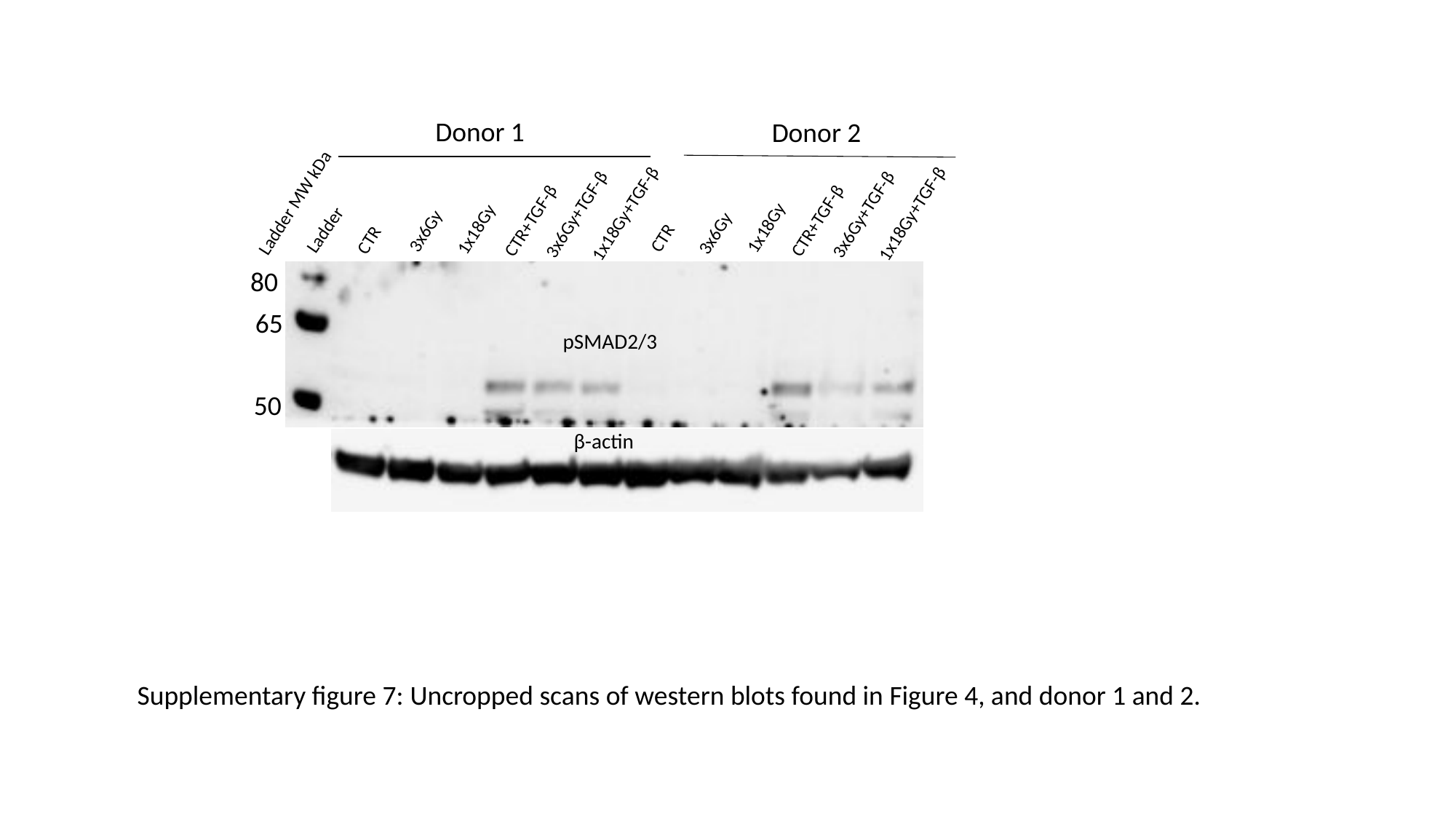

Donor 1
 Donor 2
CTR
3x6Gy
1x18Gy
Ladder MW kDa
Ladder
CTR
1x18Gy
3x6Gy
3x6Gy+TGF-β
3x6Gy+TGF-β
1x18Gy+TGF-β
1x18Gy+TGF-β
CTR+TGF-β
CTR+TGF-β
 80
 65
pSMAD2/3
 50
β-actin
Supplementary figure 7: Uncropped scans of western blots found in Figure 4, and donor 1 and 2.

## Slide 8
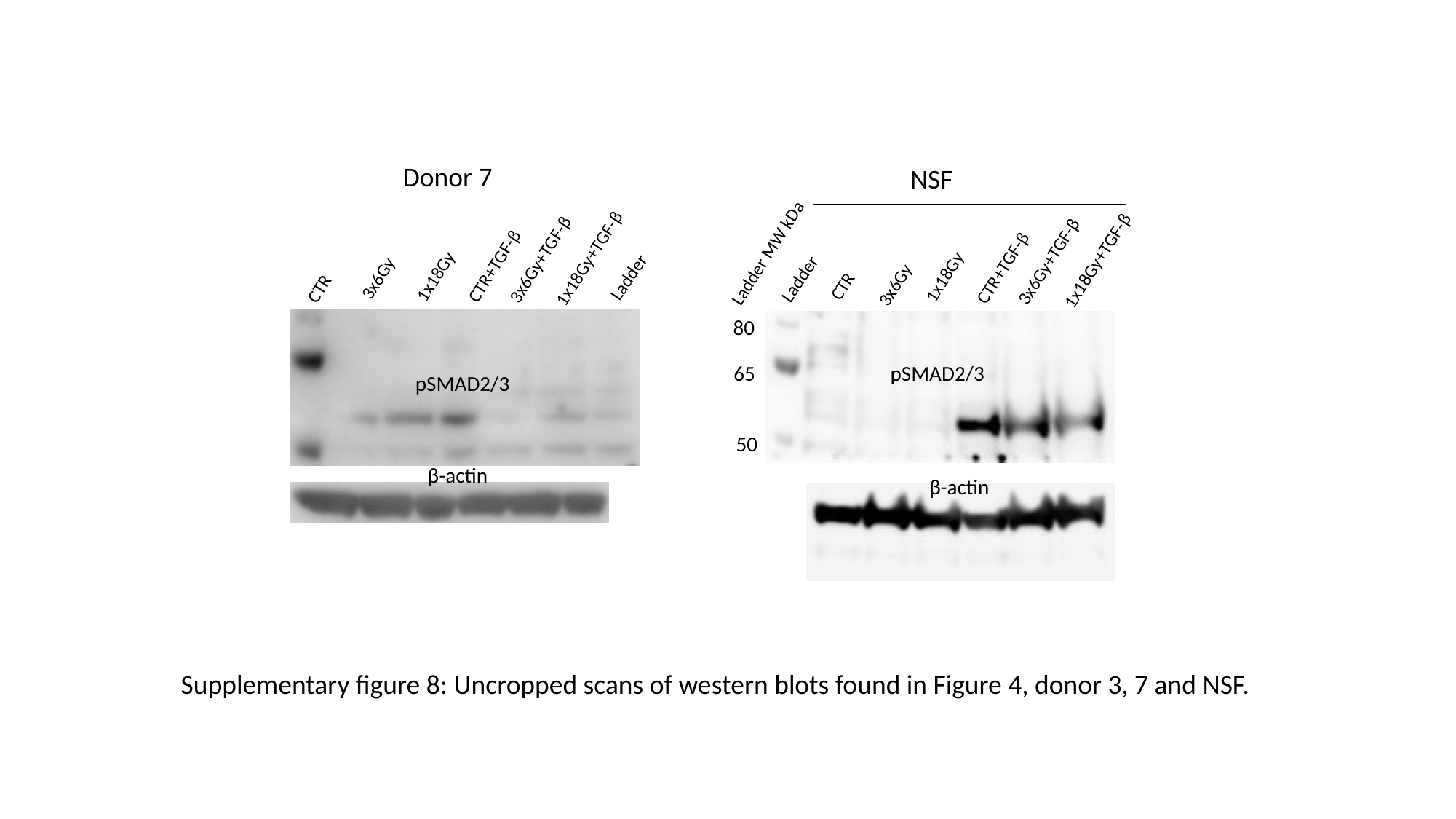

Donor 7
 NSF
3x6Gy
CTR
Ladder
1x18Gy
1x18Gy
CTR
Ladder
Ladder MW kDa
3x6Gy
3x6Gy+TGF-β
3x6Gy+TGF-β
1x18Gy+TGF-β
1x18Gy+TGF-β
CTR+TGF-β
CTR+TGF-β
 80
 65
pSMAD2/3
pSMAD2/3
 50
β-actin
β-actin
Supplementary figure 8: Uncropped scans of western blots found in Figure 4, donor 3, 7 and NSF.

## Slide 9
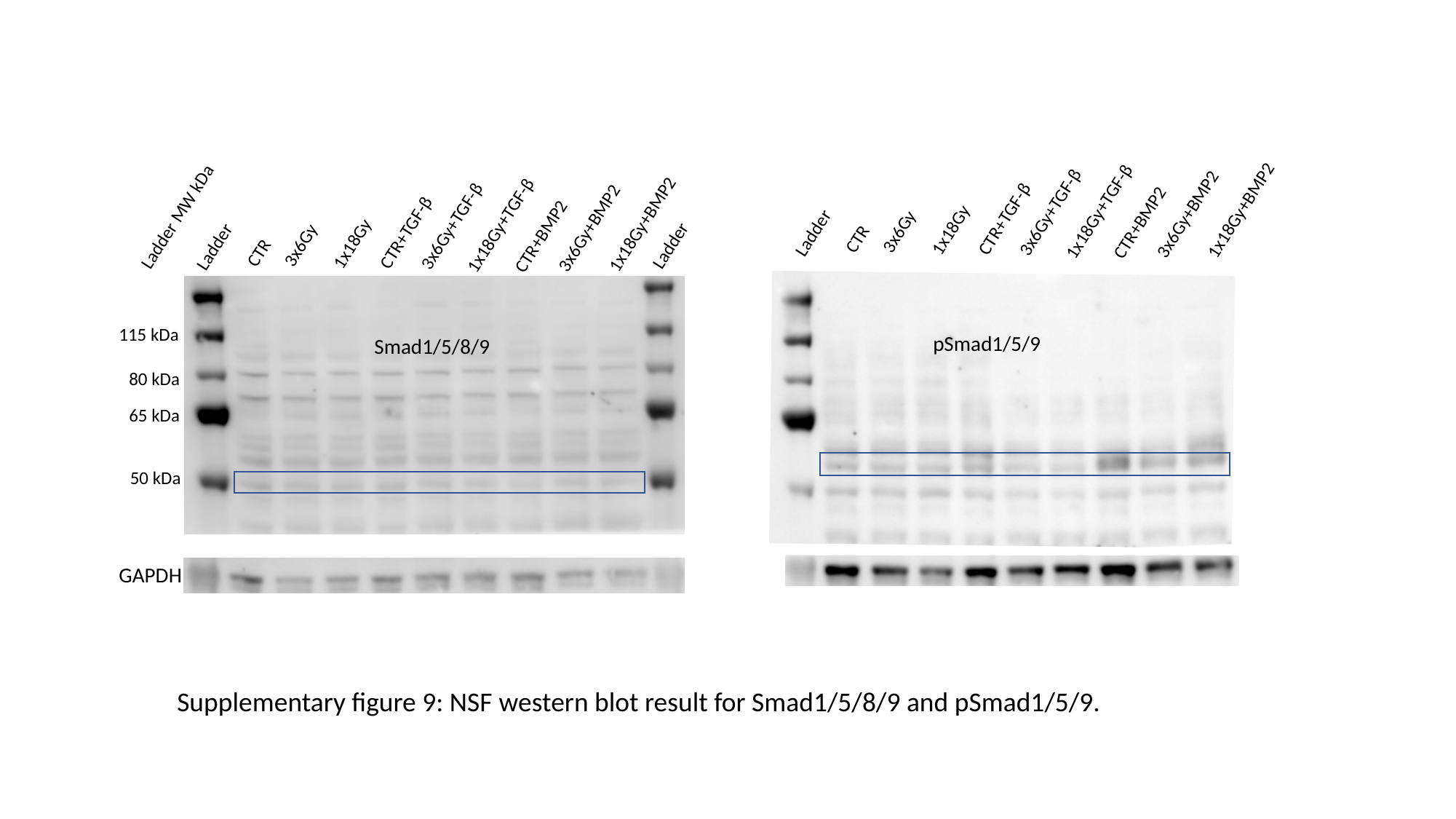

Ladder
1x18Gy+BMP2
3x6Gy+TGF-β
3x6Gy+BMP2
1x18Gy+TGF-β
Ladder
Ladder MW kDa
Ladder
CTR+BMP2
CTR+TGF-β
1x18Gy+BMP2
3x6Gy+TGF-β
3x6Gy+BMP2
1x18Gy+TGF-β
1x18Gy
3x6Gy
CTR+BMP2
CTR+TGF-β
CTR
1x18Gy
3x6Gy
CTR
115 kDa
pSmad1/5/9
Smad1/5/8/9
80 kDa
65 kDa
50 kDa
GAPDH
Supplementary figure 9: NSF western blot result for Smad1/5/8/9 and pSmad1/5/9.
